# Investigating the Mechanical Properties of Polymer Samples from Different Additive Manufacturing Processes Using Ultrasonic Phase Spectroscopy

**DOI:** 10.1089/3dp.2022.0148

**Published:** 2024-04-16

**Authors:** Philipp Eyer, Sarah Enzler, Anna Trauth, Kay André Weidenmann

**Affiliations:** Department of Materials Resource Management, Augsburg University, Augsburg, Germany.

**Keywords:** Arburg plastic freeforming, additive manufacturing, ultrasonic phase spectroscopy, nondestructive testing, injection molding, fused filament fabrication

## Abstract

Additive manufacturing processes have recently been used more frequently since they offer high design freedom and easy individualization of components. The processes have been optimized to improve mechanical performance of the manufactured parts. Nevertheless, properties of components made by means of injection molding could not be reached yet. In the study at hand, ultrasonic phase spectroscopy (UPS) is used to compare the elastic properties of acrylonitrile butadiene styrene specimens manufactured by injection molding, by fused filament fabrication, and the Arburg plastic freeforming process. UPS allows a nondestructive and prompt determination of the elastic modulus and allows evaluation of the mechanical properties in every direction in space. In the end, results of UPS are compared with properties derived by uniaxial tensile tests to validate UPS as a test method for the determination of the mechanical properties of polymers. Regardless of the manufacturing process, an approximately linear dependence of the elastic moduli on the density can be determined. Furthermore, the quasistatic properties of the injection molded samples consistently exhibit the mechanical properties of the other samples by at least 10%.

## Introduction

Additive manufacturing (AM) has a huge potential to increase the efficiency of individual production and generates ongoing development of new AM processes. During the corona pandemic in particular, AM technology aroused public interest since it was possible to manufacture medical applications, such as parts of face masks, very fast. Furthermore, it became clear to many manufacturing companies how dependency on supply chains can lead to bottlenecks. AM technology can circumvent this problem through flexible production options and decentralized production.^1^

In the beginning, AM was applicable for a few thermoplastics such as acrylonitrile butadiene styrene (ABS), polyethylene, and polylactic acid only. In recent years, the range of applicable materials increased and now covers materials such as thermosets and fiber-reinforced polymers.^2^

Polymer strands or droplets in case of the Arburg plastic freeformer are deposited layer by layer according to a prior defined slicing model. Thus, individual manufacturing of complex geometries with load capable structures is possible. In contrast, AM is fairly unsuitable for mass production due to long part production time. Furthermore, in case of fused filament fabrication (FFF), filament is necessary, which is more expensive than the granulate necessary for injection molding.^3^

Nonetheless, mechanical properties usually cannot reach the reference properties achieved with injection molding using the same material.^4,5^

Mechanical properties of parts manufactured using FFF are influenced by various parameters, as for instance, temperature of the nozzle, temperature of the printing bed, diameter of the polymer strands, as well as orientation of the strands. Influence of the temperature profile on the bonding of the strands was investigated by Vanaei et al.^6^

Improvement of the mechanical properties can be achieved by changing the orientation of the polymer strands dependent on the load. The pattern type indicates the direction in which the extrusion strands are deposited on the printing bed. To maximize tensile strength, load must be applied axially along the polymer strands (0°).^7^

The effect of different printing paths of FFF specimens on the mechanical properties has already been investigated lately. Dawoud et al stated that the raster gap is a crucial parameter, and with negative values of the gap, densities close to the density of injection molding parts can be reached. In the study, the influence of different angle layups of injection molded and fused deposition modeled parts on the tensile strength and the flexural strength was investigated.^5^

Ahn et al determined the tensile and compression strength for samples with different pattern types as well as different colors. ABS was used to manufacture the specimens using FFF and injection molding. Comparison of the results revealed that the tensile strength was mainly affected by the air gap and raster orientation. In contrast, bead width, model temperature, and color had little effect. The measured tensile strengths of the FFF samples, −45°/45° as well as 0°/90°, were between 65% and 72% lower compared with the injection molded samples. Due to the anisotropic behavior of the FFF samples, the strength depends highly on the raster direction.^8^

Ramezani Dana et al determined the influence of different layer orientations on tensile modulus and ultimate tensile strength of ABS (Terluran GP35) samples manufactured with the Arburg plastic freeformer. Highest moduli and tensile strengths were determined on samples with −45°/45° oriented layers, which were comparable to injection molded parts.^9^

Pinter et al investigated the mechanical properties of ABS samples manufactured with FFF, injection molding, and the Arburg plastic freeforming (APF) process. FFF specimens were manufactured with −45°/45° pattern type, and tensile tests, three-point flexural tests, as well as Charpy impact tests were performed. Distinct correlation between density and elastic modulus has been shown for tensile tests of all manufacturing methods. Elastic modulus decreased with the density of the samples. Same applied to the ultimate strength and the flexural modulus of the specimens. Impact of the density resulting from the process on the mechanical properties was higher than the impact of the process itself. Arburg plastic freeforming led to a more consistent density of the specimens compared with FFF.^4^

In all studies, mentioned above, destructive mechanical testing was used to determine the mechanical properties. In contrast, ultrasonic phase spectroscopy (UPS) has proven to be a more accurate and advantageous method determining mechanical properties compared with destructive testing methods.^10^ UPS is based on the measurement of the wave velocity in the material. Due to the correlation between the velocity with which the ultrasound propagates through the solid and the elastic properties, all direction-dependent components of the elastic modulus and thus the stiffness matrix can be determined by means of this material testing method.

The method is described in detail in the studies by Wanner^11^ and Lynnworth et al^12^ and was established for the characterization of metal matrix composites by Roy et al with participation of one of the authors of the study at hand. In detail, Roy et al investigated the elastic properties of interpenetrating metal/ceramic composites using UPS. By determining the complete stiffness matrix, it was possible to determine the elastic modulus, the shear modulus, as well as the Poisson's ratio. Results showed good correspondence with the predictions from a micromechanical model.^13^

Other nondestructive testing methods, such as acoustic emission,^14^ infrared thermography,^15^ or electromagnetic emission,^16^ are mainly used for the detection of cracks and other defects in critical parts such as airplane components or wind turbines. Therefore, they are essential and widely used for the health monitoring of these parts, and the methods are used regularly to spot defects at an early stage. In contrast, UPS is mainly used for the determination of the moduli of new developed materials and composites, as it is timesaving and none of the material is wasted for destructive tests.^13^

Lavrentyev and Rokhlin used UPS for determination of density, elastic moduli, and thickness of polymer coatings on steel foils.^17^ Pulse-echo ultrasound was used by Jordan et al for evaluation of elastic properties of polyethylene samples. A linear dependence between density and elastic properties could be exhibited. In addition to the density, bulk, elastic, and shear moduli were determined.^18^

To the best of the authors' knowledge, UPS has not been used for the determination of orientation-dependent elastic properties of polymers, yet. Furthermore, elastic properties of polymers from UPS measurements have not been compared with the properties determined with quasistatic experiments. Comparison of the results of destructive material testing and UPS should provide information to what extent the values differ, and which method should be selected prospectively.

## Materials and Methods

### Materials

**FIG. 1.**
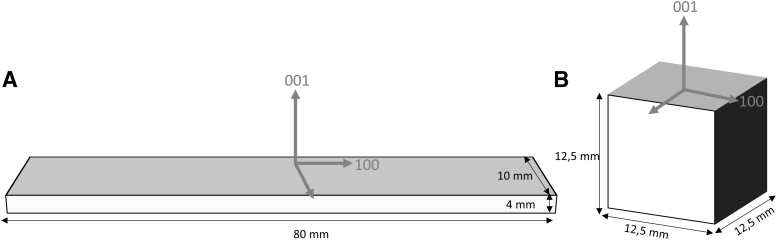
Specimen dimensions and orientation for tensile and flexural tests **(A)**; cube dimensions and orientation for UPS measurements **(B)**. UPS, ultrasonic phase spectroscopy.

All samples were manufactured using ABS granulate (Terluran GP35) provided by INEOS Styrolution Group GmbH in Frankfurt, Germany. ABS is one of the most common amorphous thermoplastics for injection molding and was the first material certified for the Arburg plastic freeformer. Furthermore, according to the literature study performed, ABS is the most common material studies of the mechanical properties of additively manufactured parts were performed on.^5–6,8,9^ Enabling comparison of the results achieved with the results found in the literature, ABS was chosen as the matrix material for all experiments. Dimensions of the specimens for flexural and tensile tests were chosen according to DIN EN ISO 178 as shown in Figure 1A.^19^

As the chosen geometry is divergent to the standard valid for tensile tests (DIN EN ISO 527-1), additional samples according to DIN EN ISO 527-1 were manufactured using the FFF to investigate the influence of the geometry on the mechanical properties.^20^

UPS measurements were carried out on cubical samples. Dimensions and orientations of the specimens, which are of importance for the UPS measurements, are shown in Figure 1B.

### Manufacturing techniques

FFF requires a filament, which was produced using a 3Devo filament extruder. For Arburg plastic freeforming as well as injection molding, the granulate could be used directly, but filament was produced first and shredded afterward using a 3devo SHR3D IT, to ensure complete comparability of the resulting mechanical properties possibly influenced by the production method. The filament was extruded with the required diameter of 1.75 mm by adjustment of temperature and ventilation. Diameter was rechecked by an optical sensor.

#### Fused filament fabrication

**FIG. 2.**
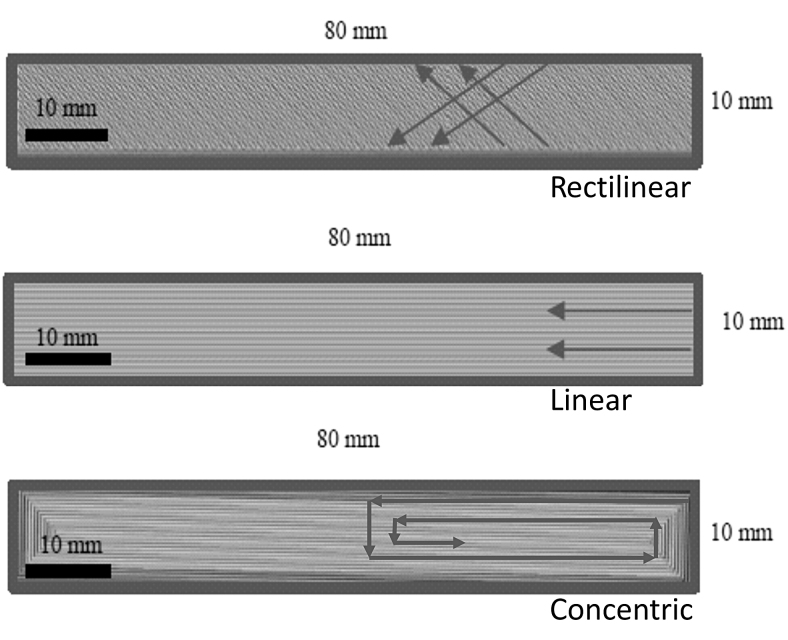
Different pattern types of FFF samples. FFF, fused filament fabrication.

FFF is a widely used extrusion-based AM process. FFF specimens were produced using a Raise3D Pro2 printer of the Raise3D Technologies, Inc., Irvine. For each printing run, the specimens were positioned at the same location in the building space, with five specimens printed at a time. To ensure meaningful comparability, matching manufacturing parameters were selected for FFF and APF samples. A nozzle temperature of 270°C, a standard layer thickness of 0.2 mm, and two shells defining the wall thickness at the level of 0.4 mm were specified. Printing orientations rectilinear (R) and linear (L), as illustrated in Figure 2, were selected for the comparison of the manufacturing methods and for investigation of the pattern type on the mechanical properties.

#### Arburg plastic freeforming

In the Arburg plastic freeforming process, small polymer droplets are applied layer by layer using a high-frequency piezo nozzle to build up the specimen. According to Arburg, mechanical properties are close to those of injection molded components.^21,22^ In addition, discharge of individual droplets results in a more homogeneous isotropy of the components.^23^ In contrast to FFF, AM on the Arburg freeformer is possible with standard granulate without prefabricated filament. For this purpose, the system has an integrated material preparation unit consisting of a plasticizing screw. Manufacturing of the samples was performed using the parameters listed in Table 1.

**Table 1.** Parameters for the Sample Manufacturing Using the Arburg Plastic Freeforming Process

Parameter	Value
Discharge coefficient	72%
Nozzle temperature	270°C
Chamber temperature	80°C
Layer height	0.2 mm
Shape factor (*d*_d_/lh)	1.34
Filling degree	96.4%

#### Injection molding

The injection molded specimens were produced on an ENGEL 120 t injection molding machine according to the settings defined in Table 2.

**Table 2.** Parameters for the Sample Manufacturing Using the Engel 120t

Parameter	Value
Nozzle temperature	230°C
Mold temperature	80°C
Injection pressure	870 bar
Dwell pressure	450 bar
Dwell time	10 s

### UPS testing

Cubic specimens are used for the UPS investigations since accurate clamping of specimens with deviating geometry between the small sensors is a problem as tilting of the specimens may lead to erroneous measurements. The cubic specimens have an edge length of 12.5 mm as shown in Figure 1B. UPS measurements were performed on an Advantest R3754A network analyzer as well as Olympus V122 longitudinal and V155 transverse broadband ultrasonic transducers. For each sample, 27 measurements were performed with a frequency range of 10 kHz and 10 MHz, and each measurement was repeated three times. Orientation of the cubes is illustrated in Figure 1B.

Continuous waves of different frequencies are transmitted through the specimen, which represents a velocity resistor for waves, resulting in a transit time difference between the input and the output signal. Phase difference of the input signal and the signal transmitted through the specimen is measured. By plotting phase difference versus frequency, the wave velocity of the sample can be determined from the slope of the graph and hence the elastic constants of the sample.

### Mechanical testing

#### Tensile tests

Tensile tests were performed on a 10 kN ZwickRoell ProLine universal testing machine. Since there is no standard for tensile testing of FFF-printed components, the test was carried out with a testing velocity of 1 mm/min in accordance with DIN EN ISO 527-1, the standard for tensile tests on cast and extruded plastic-based components.^20^ Specimen length between the clamps was 48 mm. The optical extensometer ZwickRoell videoXtens was used for exact measurement of the elongation. The tensile modulus was determined in accordance with the standard by determining the slope of the regression curve between 0.05% and 0.25% elongation.

#### Flexural tests

The flexural properties were determined using the three-point flexural test, in accordance with DIN EN ISO 178:2019-08 (method A) on a 0.5 kN zwickiLine universal testing machine from ZwickRoell GmbH & Co. KG, Ulm, Germany. Accordingly, a testing velocity of 2 mm/min was set and a span between the support clamps of 64 mm was maintained in accordance with the standard. The flexural modulus was determined by regression of the stress–strain curve between 0.05% and 0.25% elongation.^19^

## Results and Discussion

FFF and APF processes do not differ fundamentally in terms of component manufacturing. Both rely on molten polymer that is discharged according to a prior uploaded three-dimensional model. In case of FFF, polymer strands are discharged, whereas droplets are discharged in case of the APF. Material bonding of the individual layers determines the mechanical properties achieved in both manufacturing methods. The injection molding process differs significantly from the AM processes in terms of the relevant setting and sequence. Hence, other manufacturing parameters are of importance. Material properties of injection molded samples were only determined as reference values within the scope of this work.

### Tensile tests

**FIG. 3.**
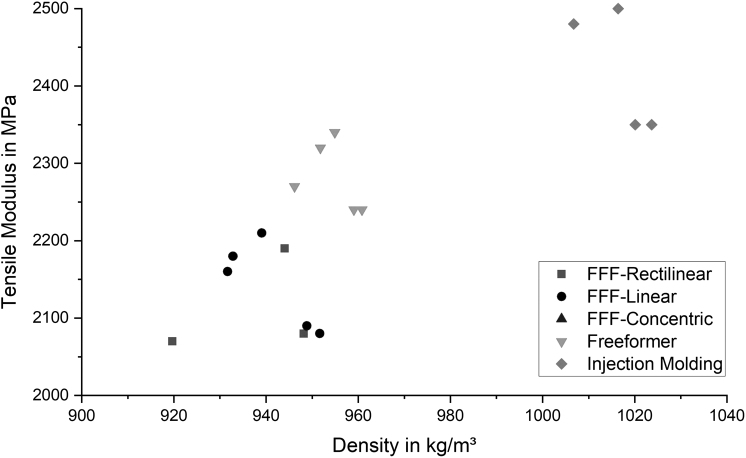
Comparison of the tensile moduli and densities for different manufacturing methods.

A clear correlation between the tensile modulus and the density of the samples can be observed as illustrated in Figure 3. Injection molded samples feature the highest tensile moduli (2442 ± 58 MPa) and the highest densities (1013.03 ± 9.3 kg/m^3^). Using the Arburg plastic freeforming process, it was possible to achieve tensile moduli, which are only 5% lower (2309 ± 44 MPa) than the moduli of the injection molded samples (2442 ± 58 MPa). The same applies to the density (954.54 ± 5.2 kg/m^3^) where the deviation is also 5%. Samples manufactured using the FFF process feature the lowest tensile moduli and the lowest densities. Tensile samples with geometric dimensions according to DIN EN ISO 527-1 achieved 1980 ± 49 MPa, which is close to the results of the FFF-R (2056 ± 82 MPa) samples manufactured the same way. Thus, influence of the specimen geometry is neglectable as it is within the standard deviation.

### Flexural tests

**FIG. 4.**
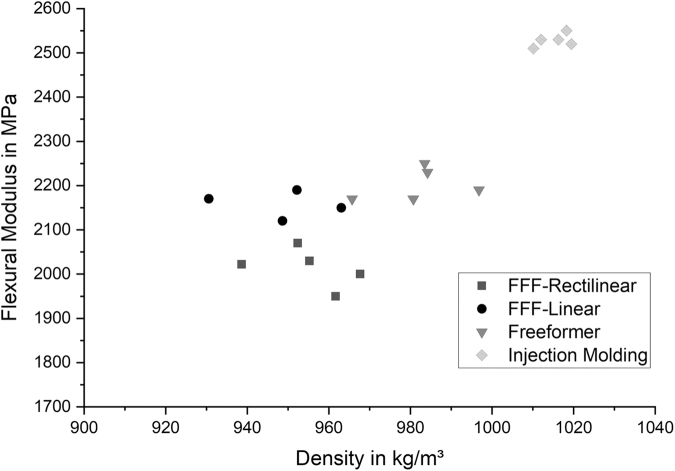
Comparison of the flexural moduli and densities for different manufacturing methods.

Similar results were achieved performing three-point flexural tests as shown in Figure 4. Injection molded samples still consist of the highest flexural modulus (2527 ± 13 MPa), followed by the samples from the Arburg freeformer process (2156 ± 55 MPa). Results of the FFF samples slightly differ from the results of the tensile tests. FFF samples consisting of a linear pattern type (compare the Tensile Tests section, Fig. 2) achieved higher flexural moduli (2154 ± 24 MPa) compared with the samples with rectilinear pattern type (2014 ± 39 MPa).

### Ultrasonic phase spectroscopy

**FIG. 5.**
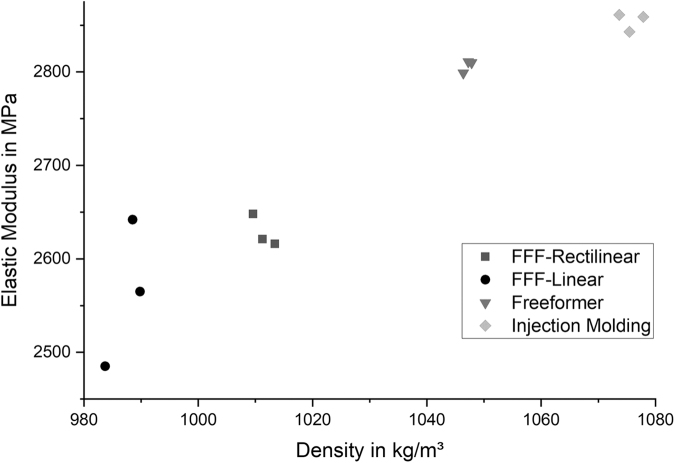
Comparison of the UPS moduli and densities for different manufacturing methods.

The UPS enables the determination of the elastic moduli in every direction in space. Consistent with the results of the destructive mechanical testing, injection molded samples show the highest moduli of elasticity. Figure 5 shows the UPS moduli of the 100 orientated samples as well as the densities for all manufacturing methods. Standard deviation of the freeformer modulus (2806.66 ± 5.44 MPa) is smaller compared to all FFF samples and even smaller than deviation of the injection molded samples (2854.33 ± 8.05 MPa).

**FIG. 6.**
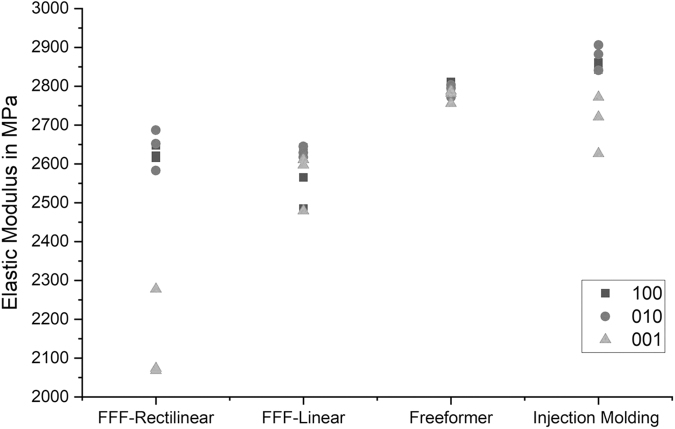
Comparison of the direction-dependent elastic moduli for different manufacturing methods.

Figure 6 exemplifies the elastic moduli for the different directions in space. At least for the 100 and 010 orientations, injection molded samples show the highest moduli, whereas the 001 orientation shows smaller values (orientations are indicated in Fig. 1). In contrast, samples manufactured with the Arburg freeformer show almost no dependency on the orientation. FFF-R samples with rectilinear-oriented polymer strands show higher moduli in the 100 and 010 orientations compared with the 001 orientation. This cannot be approved by the FFF-L samples.

### Comparison with the literature

Similar findings can be found in the literature. Rybachuk et al confirmed that the alignment of the plastic strands in the direction of the load leads to an average increase of the modulus of 40%. The lowest elastic modulus was obtained with an orientation of the strands vertically to the direction of loading.^24^ Comparison of the values is not possible due to the different manufacturing parameters chosen for each study.

In tests carried out by Pinter et al on the mechanical properties of ABS specimens, moduli of the APF and injection molding samples are in the same range as in the study at hand.^4^ Same applies to the FFF-R specimens, which is the only pattern type investigated in their study. Densities of all specimens are coincident as well within the scope of process-engineering deviations. Both studies coincide clear dependence of the elastic moduli on the density and a deviation of the tensile moduli from the flexural moduli. The deviation could be another sign for the anisotropy of the layer buildup. Study by Ozcelik et al on the variation of the manufacturing parameters of injection molded samples resulted in an average tensile modulus of 2402 MPa.^25^ Results, obtained in the present work, show an average tensile modulus of 2442 MPa, which coincides within the range of process-engineering deviations.

Investigations on the APF process by Ramezani Dana et al showed a tensile modulus of about 2200 MPa with matching print direction and similar manufacturing parameters.^9^ This coincides with the average tensile modulus of 2309 MPa determined in the present work. Tensile moduli of the APF specimens achieved, correspond on an average of 95% with the tensile moduli of the injection molded specimens. The statement of Arburg that APF allows mechanical properties close to the those of injection molded parts could be confirmed within this study.^23^ The higher moduli of the APF specimens compared to the FFF could be due to the smaller air gaps between the individual droplets due to the piezo-driven nozzle. Research results by Ahn et al showed that a negative air gap (−0.08 mm) between the plastic strands increases the density and thus the modulus.^8^ However, elastic moduli determined in the present work are in the same range.

### Discussion of the results

Increase of tensile modulus of about 34.5% was achieved by orientating the polymer strands in the direction of loading (FFF-L), compared with the FFF-R specimens. Adhesion of the polymer strands to each other is lower than the adhesion of the polymer droplets within one strand due to the manufacturing process. The specimen is created by discharging polymer strands next to each other, whereby bondings to the layer underneath and to the strands nearby are formed. Therefore, bonding behavior is dependent on the temperature of the strand itself and of the strands nearby.

Adhesion between the strands could be improved by using a printer with heated printing bed, which increases the bond to the layer underneath, as it reduces cooling of the strands already printed. Higher stiffnesses can be achieved if the sample is loaded along the polymer strands. In contrast, considering the FFF-R samples, load is applied in a direction of 45° to the polymer strands. Therefore, load is applied on the interface between the strands as well, which arouses the lower values of the stiffness for these samples as the weakest bonding is crucial for the behavior of the complete sample.

Higher standard deviation was observed throughout the samples manufactured with FFF. A possible and production-related reason could be the variation of the filament diameter, which leads to a varying material flow and ultimately favors local density differences or internal material defects. This could be solved by using FFF systems equipped with online monitoring systems to avoid over- and under-extrusion.^26^ Furthermore, the poor surface quality resulting from the FFF process can cause slippage of the specimens during tensile and flexural testing.

In addition to the problem of the stair-step effect that occurs, the poor surface quality leads to inaccurate measurement of the specimen geometry and thus to altered values of the elastic modulus as well as the geometric density. Another possible reason for the high standard deviation of the FFF specimens could be the temperature gradient arising in the layer structure. As mentioned above, in the FFF printer used for the study at hand, only the printing bed is heated, while the building space itself cannot be heated. This arouses faster cooling and thus worse bonding to the layer underneath with increasing number of layers.^27^

Elastic modulus of all FFF specimens could be further increased, for instance, by selecting smaller layer thicknesses or by printing only one sample per print run, to increase bonding of the polymer strands. If many samples are printed at once, the first layer of all samples is printed before the second layer is added to the first sample. Therefore, the more samples are printed at each run, the higher the cooling of each layer before the next layer is added, which impacts the bonding of the layers negatively.

Differences of the elastic moduli for different direction in space mainly result from the principle of layer-by-layer buildup of AM. FFF specimens show higher anisotropy of the mechanical properties than the APF specimens, which is shown in Figure 6. Besides, in case of injection molding, differences of the elastic modulus result mainly from the one-sided injection of the polymer mold, whereby the directional dependence is less pronounced compared with FFF specimens. Elastic moduli of the FFF-R specimens are significantly lower in the direction of layer buildup than parallel to the building plate as it can be seen in Figure 6. UPS results of the FFF-L samples are probably not meaningful as the pattern type is anisotropic and the UPS examination program is valid for orthotropic specimens only.^13^

The study led to coincident results of the moduli of elasticity, determined by destructive methods as well as by UPS. The UPS moduli are on average 18.5% higher than the flexural moduli and 15.9% higher than the tensile moduli. Several issues can be considered as possible reasons for the deviation of the destructive material testing. For example, excessive clamping of the specimens during tensile testing could cause premature damage of the material. In addition, flexural tests were carried out without external measuring system for displacement. Displacement of the specimens was measured by displacement of the traverse, which could falsify the results due to the flexibility of the traverse. During UPS measurements, tilting and slipping of the specimens or the UPS sensors can cause random deviations.

**FIG. 7.**
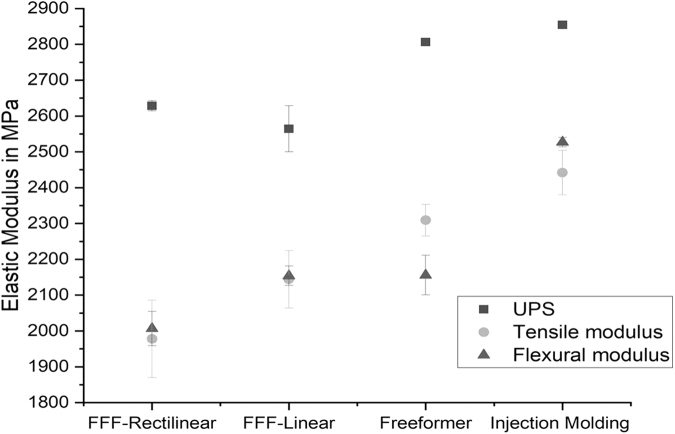
Comparison of the elastic moduli determined with different measuring methods.

However, the UPS moduli consist of lower standard deviation than the results from destructive material testing methods. Comparison of the elastic moduli of the 100 orientation measured with the UPS and the values determined using the destructive testing as shown in Figure 7 illustrates that the values of the UPS are on average 20% above the other values for each manufacturing method. It is striking that the standard deviation for UPS testing is much smaller than for the destructive testing methods. Furthermore, difference between flexural modulus and tensile modulus is more distinctive for the freeformer and the injection molded samples.

**FIG. 8.**
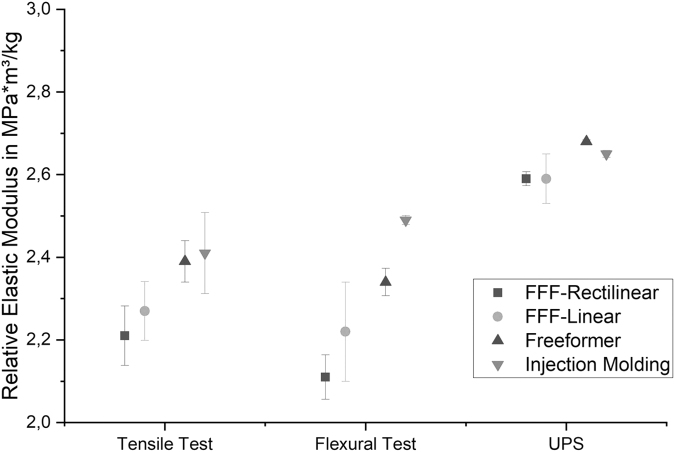
Elastic moduli standardized to the particular densities for each manufacturing method.

As the results have shown, AM of components is companied by a lower density and consequently lower elastic moduli. Figure 8 illustrates the relative elastic moduli, more precisely the elastic moduli divided by the particular density, which is an important parameter when selecting eligible materials for lightweight applications. Consistent with previous results, the highest relative modulus was achieved with injection molding, followed by the Arburg plastic freeforming process.

## Conclusion and Outlook

The aim of the study at hand was a comparison of two AM methods with conventional injection molding technology based on the resulting elastic moduli. Tensile and flexural moduli were determined by destructive quasistatic material testing. Furthermore, elastic modulus was measured using UPS. ABS specimens were manufactured additively using FFF as well as the Arburg plastic freeforming process, which is not widely used yet. Within the scope carried out in this work, an approximately linear dependence of the elastic modulus on the density could be demonstrated. The APF specimens exhibit higher densities and higher tensile and flexural moduli. Nonetheless, injection molded specimens feature the highest density and thus the highest elastic moduli. It turned out that UPS is suitable determining the direction with the highest and the lowest mechanical properties, respectively.

Deposition of polymer strands layer by layer, as it is the case in most AM processes, results in anisotropic mechanical properties as mechanical properties are significantly higher along the strands than orthogonal of the strands. With the aid of UPS, the direction-dependent modulus of elasticity of the FFF specimens could be demonstrated. In addition, a lower direction-dependent elastic modulus for the APF specimens was found. For future prospects, this study has shown that UPS is a sufficient and fast method to investigate the direction-dependent elastic modulus of additively manufactured parts. This is of high interest especially for the redevelopment of new materials as it is timesaving and material saving. If the direction-dependent modulus is determined using destructive testing, at least three samples are necessary to determine the modulus in every direction in space.

## References

[1] Equbal A, Akhter S, Sood AK, et al. The usefulness of additive manufacturing (AM) in COVID-19. Ann 3D Print Med 2021;2;100013; doi: 10.1016/j.stlm.2021.100013 38620418 PMC8074494

[2] Dudek P. FDM 3D printing technology in manufacturing composite elements. Arch Metall Mater 2013;58(4);1415–1418; doi: 10.2478/amm-2013-0186

[3] Richard HA, Schramm B, Zipsner T. Additive Manufacturing of Components and Structures: Neue Erkenntnisse und Praxisbeispiele. Springer Vieweg: Wiesbaden, Heidelberg; 2019.

[4] Pinter P, Baumann S, Lohr C, et al. Mechanical Properties of Additively Manufactured Polymer Samples using a Piezo Controlled Injection Molding Unit and Fused Filament Fabrication compared with a Conventional Injection Molding Process. Proceedings of the 29th Annual International Solid Freeform Fabrication Symposium—An Additive Manufacturing Conference, 2018; pp. 2219–2227.

[5] Dawoud M, Taha I, Ebeid SJ. Mechanical behaviour of ABS: An experimental study using FDM and injection moulding techniques. J Manuf Processes 2016;21;39–45; doi: 10.1016/j.jmapro.2015.11.002

[6] Vanaei HR, Khelladi S, Deligant M, et al. Numerical prediction for temperature profile of parts manufactured using fused filament fabrication. J Manuf Processes 2022;76;548–558; doi: 10.1016/j.jmapro.2022.02.042

[7] Es-Said OS, Foyos J, Noorani R, et al. Effect of layer orientation on mechanical properties of rapid prototyped samples. Mater Manuf Processes 2000;15(1);107–122; doi: 10.1080/10426910008912976

[8] Ahn S-H, Montero M, Odell D, et al. Anisotropic material properties of fused deposition modeling ABS. Rapid Prototyping J 2002;8(4);248–257; doi: 10.1108/13552540210441166

[9] Ramezani Dana H, Barbe F, Delbreilh L, et al. Polymer additive manufacturing of ABS structure: Influence of printing direction on mechanical properties. J Manuf Processes 2019;44;288–298; doi: 10.1016/j.jmapro.2019.06.015

[10] Blitz J, Simpson G. Ultrasonic Methods of Non-Destructive Testing, 1, ed. Chapman & Hall: London, United Kingdom; 1996.

[11] Wanner A. Elastic modulus measurements of extremely porous ceramic materials by ultrasonic phase spectroscopy. Mater Sci Eng A 1998;248(1–2);35–43; doi: 10.1016/S0921-5093(98)00524-3

[12] Lynnworth LC, Rea WR, Papadakis EP. Continuous wave transmission techniques for measuring ultrasonic phase and group velocities in dispersive materials and composites. J Acoust Soc Am 1981;70(6);1699–1703; doi: 10.1121/1.387235

[13] Roy S, Gebert J-M, Stasiuk G, et al. Complete determination of elastic moduli of interpenetrating metal/ceramic composites using ultrasonic techniques and micromechanical modelling. Mater Sci Eng A 2011;528(28);8226–8235; doi: 10.1016/j.msea.2011.07.029

[14] Romhány G, Czigány T, Karger-Kocsis J. Failure assessment and evaluation of damage development and crack growth in polymer composites via localization of acoustic emission events: A review. Polym Rev 2017;57(3);397–439; doi: 10.1080/15583724.2017.1309663

[15] Liu Y, Wu J-Y, Liu K, et al. Independent component thermography for non-destructive testing of defects in polymer composites. Meas Sci Technol 2019;30(4);44006; doi: 10.1088/1361-6501/ab02db

[16] Gade SO, Sause MGR. Measurement and study of electromagnetic emission generated by tensile fracture of polymers and carbon fibres. J Nondestr Eval 2017;36(1), doi: 10.1007/s10921-016-0386-0

[17] Lavrentyev AI, Rokhlin SI. Determination of elastic moduli, density, attenuation, and thickness of a layer using ultrasonic spectroscopy at two angles. J Acoust Soc Am 1997;102(6);3467–3477; doi: 10.1121/1.420394

[18] Jordan JL, Rowland RL, Greenhall J, et al. Elastic properties of polyethylene from high pressure sound speed measurements. Polymer 2021;212;123164; doi: 10.1016/j.polymer.2020.123164

[19] German Institute for Standardization. Plastics—Determination of Flexural Properties; 178:2019-08. Berlin: Beuth Verlag; 2019, doi: 10.31030/3030985.

[20] German Institute for Standardization e.V. Plastics-Determination of Tensile Properties: Part 1: General principles; 527-1:2019-12. Berlin: Beuth Verlag; 2012, doi: 10.31030/3059426.

[21] Neff M, Kessling O. Layered functional parts on an industrial scale. Kunststoffe Int 2014;(8);40–43.

[22] Kloke A. Droplets to the beat of milliseconds. Kunststoffe Int 2018;11(11);15–21.

[23] Kynast F. ARBURG-Kunststoff-Freiformen: freeformer-die additive Fertigungslösung von ARBURG: Arburg GmbH+Co. KG.; 2018. Available from: https://docplayer.org/108073277-Arburg-kunststoff-freiformen.html [Last accessed: February 7, 2022].

[24] Rybachuk M, Alice Mauger C, Fiedler T, et al. Anisotropic mechanical properties of fused deposition modeled parts fabricated by using acrylonitrile butadiene styrene polymer. J Polym Eng 2017;37(7);699–706; doi: 10.1515/polyeng-2016-0263

[25] Ozcelik B, Ozbay A, Demirbas E. Influence of injection parameters and mold materials on mechanical properties of ABS in plastic injection molding. Int Commun Heat Mass Transfer 2010;37(9);1359–1365; doi: 10.1016/j.icheatmasstransfer.2010.07.001

[26] Shaqour B, Abuabiah M, Abdel-Fattah S, et al. Gaining a better understanding of the extrusion process in fused filament fabrication 3D printing: A review. Int J Adv Manuf Technol 2021;114(5–6);1279–1291; doi: 10.1007/s00170-021-06918-6

[27] Vanaei H, Shirinbayan M, Deligant M, et al. Influence of process parameters on thermal and mechanical properties of polylactic acid fabricated by fused filament fabrication. Polym Eng Sci 2020;60(8);1822–1831; doi: 10.1002/pen.25419

